# Exploring the Molecular Mechanism and Biomakers of Liver Cancer Based on Gene Expression Microarray

**DOI:** 10.1007/s12253-015-9926-7

**Published:** 2015-04-25

**Authors:** Pengfei Liu, Wenhua Jiang, He Ren, Huilai Zhang, Jihui Hao

**Affiliations:** 1 Department of Lymphoma, Sino-US Center of Lymphoma and Leukemia, Tianjin Medical University Cancer Institute and Hospital, National Clinical Research Center for Cancer, Key Laboratory of Cancer Prevention and Therapy, Tianjin, China; 2 Department of Radiotherapy, The Second Hospital of Tianjin Medical University, Tianjin, China; 3 Department of Pancreatic Cancer, Tianjin Medical University Cancer Institute and Hospital, National Clinical Research Center for Cancer, Key Laboratory of Cancer Prevention and Therapy, Tianjin, China

**Keywords:** Liver cancer, Bioinformatics, Biomarker, Pathway

## Abstract

Liver cancer is one of the most common cancers worldwide with high morbidity and mortality. Its molecular mechanism hasn’t been fully understood though many studies have been conducted and thus further researches are still needed to improve the prognosis of liver cancer. Firstly, differentially expressed genes (DEGs) between six Mdr2-knockout (Mdr2-KO) mutant mice samples (3-month-old and 12-month-old) and six control mice samples were identified. Then, the enriched GO terms and KEGG pathways of those DEGs were obtained using the Database for Annotation, Visualization and Integrated Discovery (DAVID, http://david.abcc.ncifcrf.gov/). Finally, protein-protein interactions (PPI) network of those DEGs were constructed using STRING database (http://www.string-db.org/) and visualized by Cytoscape software, at the same time, genes with high degree were selected out. Several novel biomarkers that might play important roles in liver cancer were identified through the analysis of gene microarray in GEO. Also, some genes such as *Tyrobp*, *Ctss* and pathways such as *Pathways in cancer*, *ECM*-*receptor interaction* that had been researched previously were further confirmed in this study. Through the bioinformatics analysis of the gene microarray in GEO, we found some novel biomarkers of liver cancer and further confirmed some known biomarkers.

## Introduction

Liver cancer is one of the most common malignancies. It has a high morbidity and mortality, especially in sub-Saharan Africa and eastern Asia. The incidence of liver cancer has doubled or even more in the past 15 years [1]. However, the molecular mechanism of liver cancer is still largely unknown. For above reasons, an increasing number of researches on liver cancer have been conducted in recent years. Different molecular mechanism and various biomarkers related to liver cancer have been identified. Through qRT-PCR and Western blotting, JianXin et al. [2] have inferred that GOLPH3, which has higher expression level in gene and protein level of liver cancer patients compared with that of the normal population, is a new biomarker for liver cancer. Mah et al. [3] have found that the inflammation-related pathway NFkB plays an important role in liver cancer by analyzing the methylation profile of 59 liver cancer patients. Despite a great number of previous researches, molecular mechanism of liver cancer has not been fully grasped. Hence, further researches of molecular level, such as researches of gene or protein, are still needed to find out new molecular mechanism or biomarkers in an effort to improve the prognosis, diagnosis and treatment of liver cancer.

Mdr2-knockout (Mdr2-KO) mice lack the liver-specific P-glycoprotein responsible for phosphatidylcholine transport across the canalicular membrane, which may result in dysfunctional phospholipid secretion [4]. Signs of inflammation are accompanied by an increase in plasma transaminase levels and followed by enhanced connective tissue storage and fibrosis progression. As a consequence of chronic inflammation and progressing fibrosis, Mdr2-knockout mice may develop liver cancer between the ages of 12 and 15 months [5].

With the rapid growth of microarry and its implication in cancer research, a lot of genes that are related to cancers (including liver cancer) have been verified. For example, Yang et al. [6] have found that Gα12 is an important therapeutic target for liver cancer through cDNA microarray analysis. Xu et al. [7] have verified, through microarray and RT-PCR technology, the role of CXCL5 in liver cancer migration and invasion.

In this research, by analyzing the gene expression microarray of liver cancer in GEO database, we further confirmed the molecular mechanism and some biomarkers of liver cancer that had been investigated previously. Moreover, genes, which had not been researched but also had a great importance to liver cancer, were also included in this research. Also, most of the enriched GO terms and KEGG pathways of those genes were related to liver cancer, especially cell cycle, immune response, inflammatory response, *pathways in cancer*, *MAPK signaling pathway*, *Cell adhesion molecules* and etc. In conclusion, our finding can improve our understanding of liver cancer and provide potential therapeutic targets for further studies.

## Materials and Methods

### Gene Expression Microarray Data

In this study, the gene expression microarray data set GSE4612 was downloaded from the Gene Expression Omnibus (GEO, http://www.ncbi.nlm.nih.gov/geo/). GSE4612 [8] is a gene expression profile data including six Mdr2 knockout (Mdr2-KO) mutant mice samples(3-month-old and 12-month-old) and six control mice samples(3-month-old and 12-month-old). The platform of this microarray data is GPL339 [MOE430A] Affymetrix Mouse Expression 430A Array.

### Preprocessing of the Microarray Data

Unwanted noise of the raw microarray data was filtered out in the preprocessing stage. The normalization of raw data and background correction was conducted via affy[9] package in R. Moreover, multiple probes that corresponded to one gene symbol were summarized–taking the average expression values of those probes as the expression value of this gene. There were a total of 22,690 probes in the microarray and 13,687 gene symbols that had no duplicate before and after preprocessing.

### Get the Differentially Expressed Genes

After the preprocessing, the critical step was to get the differentially expressed genes (DEGs) between the case samples and the control samples. The tool used in this study was the limma [10] package in R. *t*-test was conducted on the gene expression values between case samples and control samples and the genes with *P* value < 0.05 and |log_2_(fold change)|>1 were selected out. According to those criteria, in the first step, the DEGs between the case samples and the control samples were selected out from 3-month-old mice and 12-month-old mice respectively, then the overlapped genes between those two list DEGs were selected out. The heatmap of the overlapped DEGs was obtained through gplots package in R to visualize their expression value in different samples.

### GO Enrichment and KEGG Pathway Analysis of the DEGs

After getting the DEGs, GO enrichment and KEGG pathway analysis of the DEGs were conducted. Here, the tool used in this study was DAVID (http://david.abcc.ncifcrf.gov/) (Database for Annotation, Visualization and Integrated Discovery). It could be used to do functional annotation for a list of genes, gene functional classfication or gene ID conversion. In this study, the module used in this study was the functional annotation. First, we submitted the DEGs list into the database and selected Mus musculus in species column. Finally, the GO terms and the KEGG pathways with *P* value smaller than 0.05 and at least five genes were selected out as the enriched function of DEGs.

### Construct the PPI Network of DEGs

To further investigate the molecular mechanism of liver cancer, PPI network of the DEGs was constructed through STRING database (http://www.string-db.org/). STRING is a database that infers the interaction between genes through analyzing the genomic data that comes from different sources, such as high-throughput experiments, co-expression data and the previous data and etc. Also, it has a unique scoring framework which assigns the interaction an integrated score to represent its confidence through combining the score of the different sources. Here, we selected the gene-gene interactions, whose integrated scores were bigger than 0.4 (the default threshold in the STRING database), to construct the PPI network and Cytoscape [11] was used for visualization.

### Select the Core Gene in the Network

To select core genes (the genes that might be more likely involved in liver cancer) from PPI network, we analyzed the topological structure of the network and obtained the degree (the number of genes that directly interact with the gene) of each gene. Here, we selected the genes whose degree is beyond 10 as the core genes in the network.

## Results

### Differentially Expressed Genes (DEGs)

**Fig. 1:**
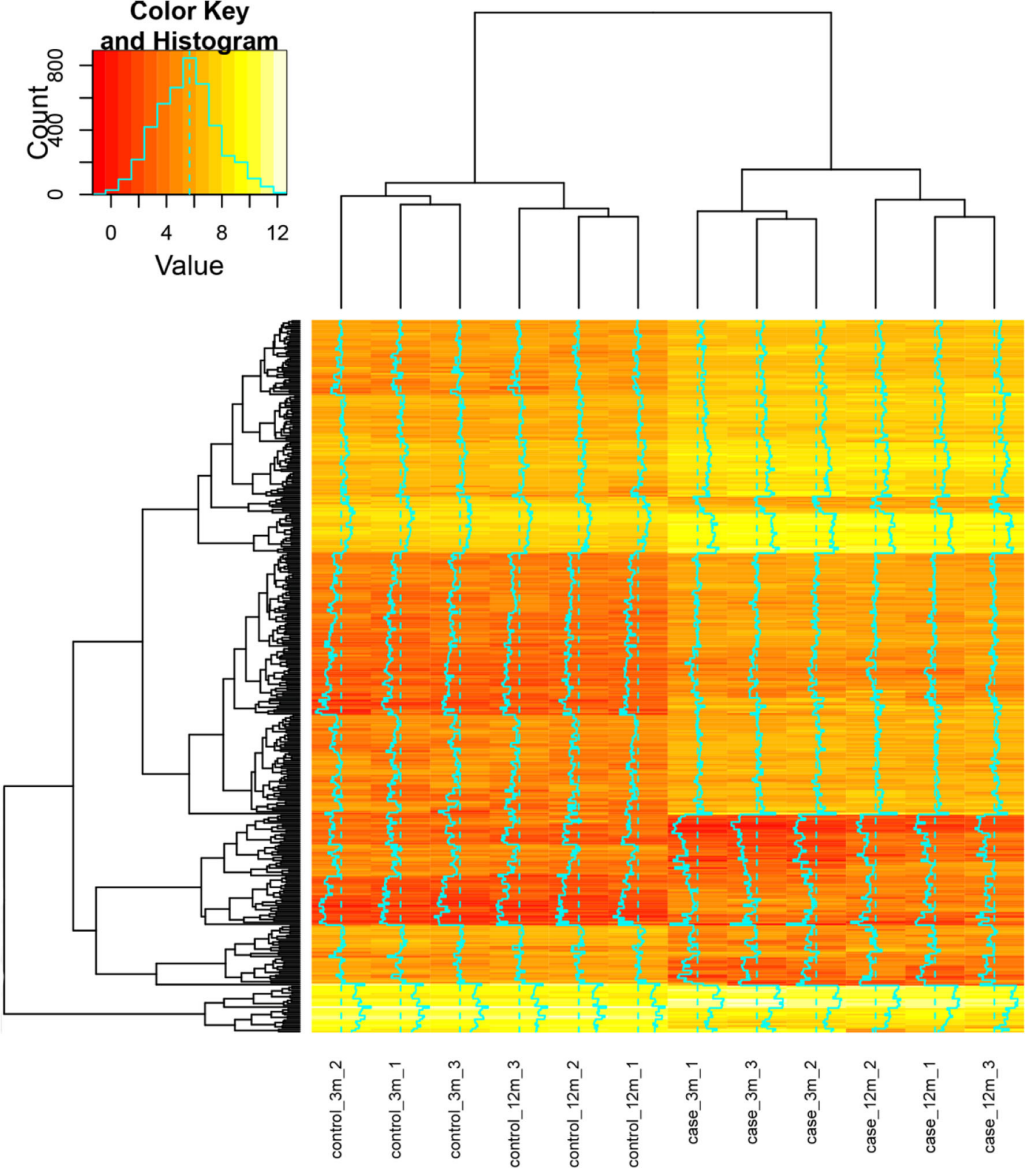
The heatmap of the DEGs. In the figure ‘control_3m_n (*n* = 1, 2, 3)’ is the 3-month-old mice in control samples; ‘control_12m_n (*n* = 1, 2, 3)’ is the 12-month-old mice in control samples; ‘case_3m_n (*n* = 1,2,3)’ is the 3-month-old mice in case samples; ‘case_12m_n (*n* = 1,2,3)’ is the 12-month-old mice in case samples

There were 1898 DEGs in the 3-month-old mice and 864 DEGs in the 12-month-old mice between the case samples and control samples. A total of 380 overlapped DEGs between those two DEG lists were identified. From the heatmap (Fig. 1), we could get that the gene expression of Mdr2 knockout samples were distinguished from the control samples, meanwhile, the gene expression of 3-month-old samples were distinguished from the 12-month-old samples, indicating that obvious differences existed in these groups.

**Table 1:** The top 10 enriched GO terms of DEGs, which were sorted by *P* value in ascending

Category	GOID	GO name	*P* value	Gene number
CC	GO:0005578	proteinaceous extracellular matrix	2.74E-08	25
CC	GO:0005576	extracellular region	5.41E-08	70
CC	GO:0031012	extracellular matrix	5.82E-08	25
CC	GO:0044421	extracellular region part	7.71E-08	42
BP	GO:0006955	immune response	1.98E-05	26
BP	GO:0006952	defense response	2.54E-05	25
BP	GO:0007155	cell adhesion	5.24E-05	28
BP	GO:0022610	biological adhesion	5.37E-05	28
MF	GO:0004197	cysteine-type endopeptidase activity	2.65E-04	8
BP	GO:0006954	inflammatory response	2.88E-04	15

### Enriched GO Terms and KEGG Pathways of DEGs

In this study, a total of 128 enriched GO terms and 23 KEGG pathways were obtained. The top 10 enriched GO terms of the DEGs according to *P* value were shown (Table 1). Table 1 indicated that the main enriched GO terms was the biological process of cell, such as cell adhesion, regulation of cell growth, regulation of cell cycle. Besides the cell biological process, there were also some enriched GO terms related to immune response, inflammatory response and etc.

**Fig. 2:**
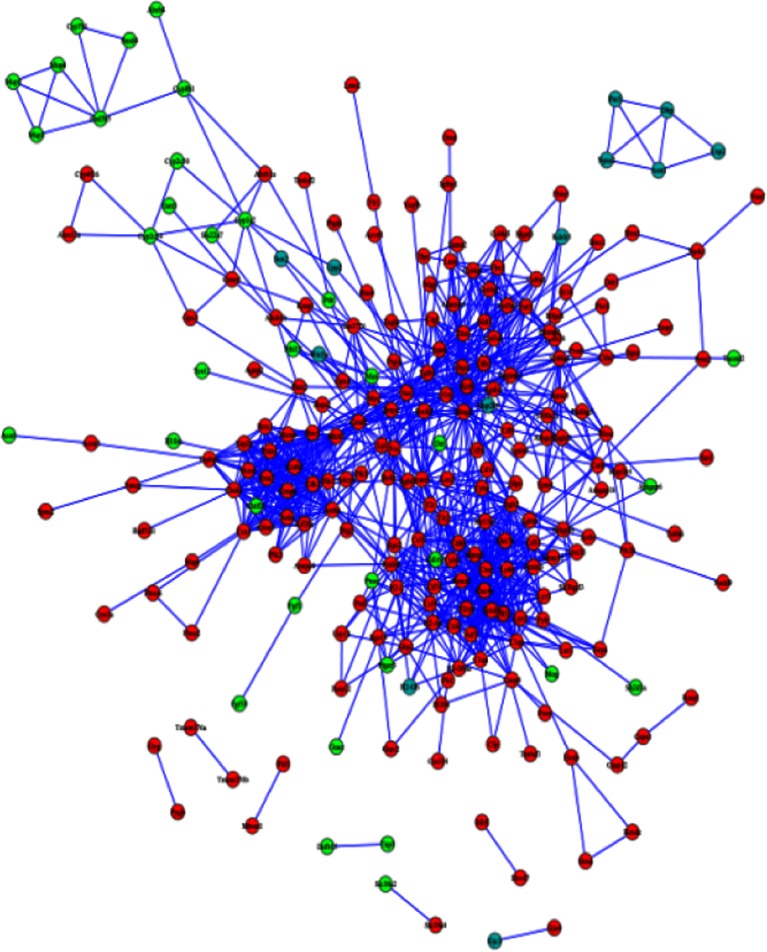
The PPI network of the DEGs. The network contains 244 nodes and 1053 edges. The 198 crimson nodes are the genes that have higher expression values in the case samples compared with the control samples in both 3-month-old mice and 12-month-old mice; the 34 bright green nodes are the genes that have lower expression values in the case samples compared with the control samples in both 3-month-old mice and 12-month-old mice; the 12 blue nodes represent the genes that have contradictory expression trend between case samples and control samples in 3-month-old mice and 12-month-old mice

The enriched KEGG pathways of the DEGs were shown in Table 2. A few enriched KEGG pathways were directly related to cancer, such as *Pathways in cancer*, *Small cell lung cancer*, *Bladder cancer*. What’s more, it was possible that other pathways had an important influence on the progression of cancer via some biological process, such as Toll-like receptor signaling, EMC-receptor interaction, MAPK signaling pathway and etc. The KEGG pathways and their corresponding gene number were shown in Fig. 2.

**Table 2:** The KEGG pathways enriched in DEGs

Category	Pathway name	Gene number	*P* value
KEGG_PATHWAY	mmu04612:Antigen processing and presentation	10	3.22E-04
KEGG_PATHWAY	mmu05416:Viral myocarditis	10	4.11E-04
KEGG_PATHWAY	mmu05200:Pathways in cancer	18	0.001908471
KEGG_PATHWAY	mmu04110:Cell cycle	10	0.003677215
KEGG_PATHWAY	mmu04512:ECM-receptor interaction	8	0.003769465
KEGG_PATHWAY	mmu00590:Arachidonic acid metabolism	8	0.003769465
KEGG_PATHWAY	mmu04514:Cell adhesion molecules (CAMs)	11	0.003969349
KEGG_PATHWAY	mmu05320:Autoimmune thyroid disease	7	0.007639543
KEGG_PATHWAY	mmu05310:Asthma	5	0.007822698
KEGG_PATHWAY	mmu04672:Intestinal immune network for IgA production	6	0.00947486
KEGG_PATHWAY	mmu04620:Toll-like receptor signaling pathway	8	0.009821668
KEGG_PATHWAY	mmu04010:MAPK signaling pathway	14	0.011527368
KEGG_PATHWAY	mmu05332:Graft-versus-host disease	6	0.012714159
KEGG_PATHWAY	mmu05330:Allograft rejection	6	0.012714159
KEGG_PATHWAY	mmu05222:Small cell lung cancer	7	0.016578151
KEGG_PATHWAY	mmu04940:Type I diabetes mellitus	6	0.017724229
KEGG_PATHWAY	mmu05219:Bladder cancer	5	0.018106115
KEGG_PATHWAY	mmu04510:Focal adhesion	11	0.021349818
KEGG_PATHWAY	mmu04670:Leukocyte transendothelial migration	8	0.024856275
KEGG_PATHWAY	mmu04912:GnRH signaling pathway	7	0.029651851
KEGG_PATHWAY	mmu00480:Glutathione metabolism	5	0.036428615
KEGG_PATHWAY	mmu04710:Circadian rhythm	3	0.038159626
KEGG_PATHWAY	mmu05322:Systemic lupus erythematosus	7	0.038193344

### PPI Network of the DEGs and Core Genes in the PPI Network

**Fig. 3:**
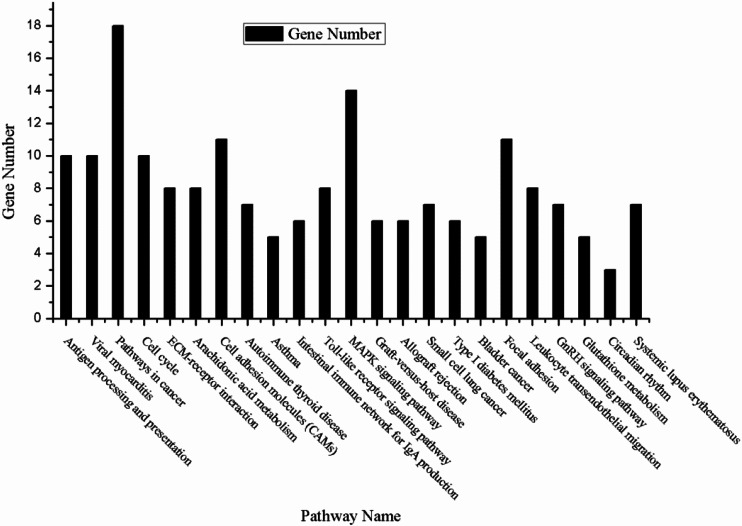
The KEGG pathway and their corresponding gene number

The PPI (Fig. 3) network contained 244 nodes and 1053 edges. The nodes represented the DEGs and the edges represented the interactions between the DEGs. A great number of genes of higher degree, which were the core genes in the PPI network, might relate to liver cancer more closely. The core genes and their corresponding degree were shown in Table 3. Among those core genes, *Ctss* and *Tyrobp* had the highest degree and there were 28 genes whose degree was beyond 20.

**Table 3:** The core genes and their corresponding degree

Gene	Degree^a^	Gene	Degree	Gene	Degree	Gene	Degree
Lum	10	Fbn1	15	Mcm5	19	Cdh1	24
Tpm4	10	Itga6	15	Cdca5	19	Ccnd1	25
Lyz1	10	Lgals1	15	Tlr2	20	Jun	25
Gbp2	10	S100a6	15	Serpinh1	20	Fos	25
Max	11	Fermt3	16	Lyz2	20	Cd86	25
Gm3776	11	P2ry6	16	Cd53	20	Rmcs2	25
Col4a2	11	Ccnf	17	Clec4n	20	Birc5	25
Ctgf	12	Chtf18	17	H2-Aa	20	Ccnb2	25
Arhgdib	12	C1qa	17	Prim1	20	Ccl5	26
Cd14	12	Anln	17	Mcm6	20	Ly86	26
H2-L	12	Col5a2	17	Rrm2	20	Icam1	27
Cd74	12	Cybb	17	Kif20a	20	Mki67	27
F2r	12	Col3a1	18	Slc15a3	21	Aurka	27
Efemp2	13	Dcn	18	Clec7a	22	Itgb2	27
Cyba	13	C1qc	18	Plk1	22	Mmp2	28
Rock2	13	Myc	18	Chek1	22	Cdk1	30
Ccl9	13	Psmb8	18	Cd52	22	Vim	31
Topbp1	14	Fstl1	19	Bgn	23	Tyrobp	37
Col4a1	14	Ccl6	19	Aif1	24	Ctss	37
Nis1	14	Rrm1	19	Mpeg1	24		
Cenpq	15	Top2a	19	Tgfb1	24		

^a^The number of genes that directly interact with the genes in the PPI network

## Discussion

Although researchers have made considerable efforts in disclosing the mechanisms of liver cancer,current understanding of the genetic alterations associated with the progression of liver cancer has not yet to be elucidated. In this study, we conducted genome-wide gene expression analysis by a high throughput method to identify the DEGs from liver cancer compared with normal liver tissues. Here, a total number of 380 overlapped DEGs from original dataset of two groups (3-month-old group and 12-month-old group) were identified, including 289 overexpressed genes, 66 down-regulated genes and 25 genes that had contradictory expression trend.

GO analyses revealed that the significant ontology categories included immune response, cell adhesion, inflammatory response and so on. Immune effector process, nuclear division, cell division, mitotic cell cycle and positive regulation of cellular component organization were obviously overrepresented in the up-regulated genes according to the functional enrichment analysis. In the immune response, for example, TLR2 could enhance ovarian cancer stem cell self-renewal and eventually promote tumor repair and recurrence [12]. ICAM-1 is a transmembrane glycoprotein in the immunoglobulin superfamily, which participates in oral cancer progression and induces macrophage/SCC-cell adhesion [13]. Ciftci et al. [14] indicated that serum TGFB1 level might be elevated in breast cancer patients and had a favorable prognostic value. CDH1, involved in cell adhesion, can code the adhesion protein E-cadherin that plays a central part in the process of epithelial morphogenesis [15]. CCL5 belongs to the CC-chemokine family and plays a pivotal role in the invasion and metastasis of human cancer cells. Huang et al. reported that CCL5 stimulation could increase lung cancer migration via induced phosphorylation of the p85α subunit of PI3K and serine 473 of Akt [16].

DEGs were then used in KEGG pathway analyses and 23 pathways were screened out, such as Cell adhesion molecules, Toll-like receptor signaling, EMC-receptor interaction, MAPK signaling pathway and etc. Previous researches reported that most of these pathways were involved in cancer progression. The immune system played a critical role in body defense system, and the dysfunction of immune system might result in cancer. Stimulation of various Toll-like receptors induced specific patterns of gene expression, which resulted in the activation of innate immunity and the development of antigen-specific acquired immunity [17]. Moreover, MAPK signal molecules participated in the amplification and specificity of the transmitted signals that finally activated a number of regulatory molecules in the cytoplasm and the nucleus to initiate cellular processes such as proliferation, differentiation, and development [18].

Furthermore, the topological structure analysis of PPI network suggested that Ctss, Tyrobp, Vim, Cdk1 were the top 4 core genes, which might be potential therapeutic targets for future research. Cathepsin S (Ctss), a key enzyme in major histocompatibility complex class II (MHC-II) mediating antigen presentation, might be involved in malignant progression of lung cancer [19]. CD47 positive liver cancer cells preferentially secreted cathepsin S (CTSS), which regulated liver tumor-initiating cells through the CTSS/protease-activated receptor 2 (PAR2) loop [20]. Shabo indicated that Tyrobp (DAP12) in breast cancer was associated with an advanced tumor grade and higher rates of skeletal and liver metastases [21, 22]. Costa reported that Vim could associate with GDF15 and TMEFF2 to predict bladder cancer [23].

Overall, with a microarray data set from the GEO database, a range of DEGs were obtained in liver cancer and normal tissues. These genes might be functionally relevant to pathogenesis of liver cancer. Functional analysis revealed mitotic cell cycle, proteinaceous extracellular matrix and MAPK signaling pathway participated in biological processes as the significant items for liver cancer. These results could provide a valuable data base for further investigation of liver cancer research. Of course, further experiments are still needed to further confirm the potential function of these genes.
